# Factors contributing to Korean road accidents based on insurance records

**DOI:** 10.1371/journal.pone.0340872

**Published:** 2026-01-29

**Authors:** Seongkyun Cho, Chanwoo Park

**Affiliations:** 1 Railway Safety Policy Division (2023), Ministry of Land, Infrastructure, and Transport of the Republic of Korea, Sejong, Republic of Korea; 2 System Safety Research Department (2021), Korea Railroad Research Institute, Uiwang, Republic of Korea; Tsinghua University, CHINA

## Abstract

This study aims to identify the key factors contributing to the severity of road accidents in Korea, analyzing more than 3,000 motor vehicle insurance records using the generalized ordered logit model (GOLogit). The model addresses the limitations of the parallel regression assumption, which ignores the differences between adjacent discrete levels of injury severity. The variable “Vehicle type (including pedestrian) with less liability”, which has been rarely examined in previous studies, demonstrated that individuals in the less responsible and more vulnerable position tend to suffer more severe injuries in South Korea. Consistent with this, the GOLogit estimates showed particularly high log-odds for severe injuries among pedestrians (4.912) and non-motorized cyclists (4.746), while speed-limit violations substantially increased the likelihood of fatal outcomes (2.456). In contrast, population density exhibited a protective effect, reducing injury severity (scaled log-odds = −1.055). This pattern is similar to broader societal trends, where economically disadvantaged regions tend to experience more severe traffic-related injuries. Specific road structures, such as the traditional right-angled crossroads, access roads to arterial roads, speedbumps on curved roads, and junctions between motor vehicle roads and sidewalks, pose significant safety challenges. Based on these findings, government policies on road safety should emphasize lowering the speed limits in residential areas, expanding the implementation of international pedestrian protection safety standards, and investing equitably in the safety of poor, low-population-density regions and older adults.

## Introduction

Korea’s road transport safety has not kept pace with its economic development as a member of the Organisation for Economic Co-operation and Development (OECD). In particular, pedestrian safety in Korea ranks among the worst in the OECD, despite a passenger fatality rate close to the average. The pedestrian fatality rate is notably higher among individuals aged over 65 [1]. According to the United Nations, Korea is classified as an aging society and is expected to become a super-aged society by 2026 [2]. Given the vulnerability of pedestrians and the growing elderly population, Korea presents a unique case that necessitates more integrated and structured research to improve road safety.

Effective road safety and traffic policy should be grounded in a comprehensive analysis that integrates regulatory and enforcement frameworks, persistent deficiencies in the transport system, recurrent patterns of road user behavior, and relevant socioeconomic conditions. However, comprehensive analyses encompassing all these dimensions remain scarce. Some researchers have undertaken partial integrated approaches. For instance, Chung and Won (2012) considered accident types, road user behaviors and characteristics, structural features of roads, and vehicle types as key contributors to road accidents in Korea [3]. Kim et al. (2014) analyzed traffic accidents using variables such as vehicle kilometers traveled, the proportion of migrants and individuals under 15 years of age, the number of bus and metro stations, the proportion of median-exclusive bus lanes, income levels, and school density [4]. Rolison et al. (2018) incorporated diverse factors based on data collected from multiple sources (e.g., police officers, the general public, and accident records) and demonstrated how a single factor can influence perceptions of accident likelihood across various groups [5].

However, none of these studies has simultaneously accounted for a complete set of factors, including accident types, rule violations, road geography, vehicle type, socioeconomic characteristics, and injury severity. Moreover, they do not address the limitations posed by restricted data access, as national police departments often provide only partially censored datasets. Building on these gaps, this study aims to overcome such limitations, as detailed in the Data management section. Furthermore, it integrates the concept of relative liability of the involved party with an advanced logit modeling approach.

The contributions of this research are as follows. First, we introduce the concept of the relative liability of involved parties into injury severity analysis—a factor rarely incorporated in previous studies. Second, we apply a generalized ordered logit model to address the limitations of the parallel regression assumption, thereby improving the model’s explanatory power. Third, we compile a uniquely extensive dataset that encompasses human, vehicular, road, and environmental factors, enabling a more holistic analysis than prior studies.

The remainder of this paper is organized as follows. The “Literature review” section examines previous research on the severity of traffic accidents. The “Data management” section describes the data sources, variable definitions, and modeling methodology. The “Calculations” section presents the analysis results. The “Results” section discusses the findings in the context of existing literature and policy implications. Finally, the “Conclusion” section summarizes the key takeaways.

Beyond road traffic, similar multi-factor interactions among human behavior, environmental conditions, and operational contexts arise in other safety-critical transportation domains, such as railroad operations. The multi-factor framework used in this study provides a transferable structure for examining such risk mechanisms. That is why this study is part of the “Development of Technology to Analyze, Evaluate, and Prevent Human Errors of Railroad Workers”—an R&D project funded by the Korean government.

## Literature review

Recent studies examining the relationship between injury severity and its associated factors have employed modified versions of ordered logit/probit models to address the inherent limitations of the parallel regression assumption in conventional models. These modified approaches include nested ordered logit regression, combinations with the multinomial logit model, and random parameterized ordered logit regression. However, these alternatives cannot fully resolve the assumption that the coefficients for a discrete variable in an ordered logit model are equivalent to those for a continuous variable in linear regression [6,7].

For example, Tay et al. (2008) used conventional logistic models to identify the circumstances under which a culpable driver was likely to commit a “hit-and-run” offense. The factors examined included the time of the accident, geographic features of the road at the accident site, types of nearby buildings, vehicle types involved, and drivers’ ethnicity and age [8]. Abdel-Aty (2003) applied ordered probit models to analyze driver injury severity across different crash locations, such as roadway sections, signalized intersections, and toll plazas [9]. His analysis demonstrated that several roadway and driver characteristics significantly influenced injury severity, underscoring the usefulness of ordered-response models in capturing differences across severity categories [9]. This finding indirectly suggests that the “strict parallel lines assumption” or “proportional odds assumption” may limit a model’s explanatory power. In other words, lower injury severity levels may be nested within higher severity levels, making it inappropriate to assume equivalent differences across severity categories [9].

Rezapour and Ksaibati (2018) analyzed truck crashes based on violation types, driver characteristics, and conditions at the accident locations using an ordered logit model combined with a multinomial logit model. In their analysis, the ordered logit model was used to identify factors contributing to truck crashes, while the multinomial logit model was employed to determine categorical conditions influencing rule-violation patterns [10]. Lee et al. (2018) examined the influence of vehicle type on injury severity using a flexible mixed multinomial logit model combined with a fractional logit model, where the dependent variable represented the proportion of crashes per vehicle type within a zone, and the independent variables included socioeconomic factors, traffic characteristics, land use attributes, and commuting features [11].

Anastasopoulos and Mannering (2010) employed mixed parametric models to analyze highway crash data from Indiana, wherein the model adjusted logit probabilities based on parameter density [12]. Similarly, Moorea et al. (2011) applied both multinomial and mixed logit models and found that the variables most significantly influencing injury severity varied depending on whether the crash occurred at an intersection [13]. Lee et al. (2018) and Wua et al. (2014) used mixed logit models to analyze factors contributing to pedestrian injury severity and vehicle crashes, respectively [11,14]. Savolainen et al. [15] identified several challenges associated with traffic safety analyses using logit and probit models, including crash underreporting, omitted variable bias, within-crash correlations, and small sample sizes. They emphasized the importance of incorporating data on both the more and less responsible parties in a crash to address within-crash correlation, noting that most studies focus only on the most severely injured party. To overcome these limitations, they recommended the use of models such as the Bayesian hierarchical binary logit model, bivariate or multivariate binary probit model, heterogeneous choice model, or generalized ordered logit model [15].

Wang et al. (2023) applied latent class clustering to segment motorcycle crash scenarios and subsequently estimated injury severity within each class using a partial proportional odds model [16]. This modeling strategy allowed them to capture heterogeneity in the effects of road design and control features on motorcycle crash outcomes. Table 1 of their study provides a helpful overview of the variable groups considered in prior research, and it informed the overall structure of our analysis, which incorporates 27 regulatory violations, 18 accident types, 19 roadway characteristics, and several socioeconomic indicators.

**Table 1:** Selected variables.

Independent variable	Obs.	Mean	Std. Dev.	Min	Max	Category
Single-vehicle crash with building	3,370	0.025	0.157	0	1	Vehicle types with relative liability
Crash with another vehicle parked	3,370	0.033	0.178	0	1	
Bus (more responsible)	3,370	0.222	0.415	0	1	
Two-wheeler (more responsible)	3,370	0.055	0.228	0	1	
Vehicle-to-vehicle crash at a crossroad	3,370	0.325	0.468	0	1	
Truck (more responsible)	3,370	0.055	0.229	0	1	
Jaywalker (more responsible)	3,370	0.019	0.137	0	1	
Bus (less responsible)	3,370	0.122	0.328	0	1	
Truck (less responsible)	3,370	0.043	0.203	0	1	
Two-wheeler (less responsible)	3,370	0.096	0.295	0	1	
Bike without a motor (less responsible)	3,370	0.023	0.149	0	1	
Agricultural machines (less responsible)	3,370	0.005	0.069	0	1	
Pedestrian (less responsible)	3,370	0.050	0.219	0	1	
Jaywalker (less responsible)	3,370	0.048	0.214	0	1	
Passenger (less responsible) (vehicle occupant, excluding driver)	3,370	0.157	0.364	0	1	
At a crosswalk without a light	3,370	0.018	0.131	0	1	Infrastructural types
At a junction of several roads	3,370	0.007	0.081	0	1	
At a junction of a driveway and a non-driveway	3,370	0.004	0.060	0	1	
At a crosswalk without a light and near a crossroad	3,370	0.020	0.140	0	1	
Number of lanes on the road	3,370	3.076	1.067	1	5	
Violation of the speed limit	3,370	0.007	0.082	0	1	Violation types
Violation of slowing down or stopping	3,370	0.038	0.192	0	1	
Violation of the minimum safe distance	3,370	0.016	0.127	0	1	
Crossing the centerline	3,370	0.060	0.237	0	1	
Not adhering to the priority order	3,370	0.167	0.373	0	1	
Failure to maintain a watch for safe driving	3,370	0.294	0.456	0	1	
Sudden stop or start	3,370	0.022	0.148	0	1	
Driving drunk or asleep and crossing the centerline	3,370	0.002	0.049	0	1	
Age	3,370	48.120	13.387	0	92	Socio-economic variables
Population density	3,370	6856.8	7637.3	4.864	40581.6	
Number of high schools per dong	3,370	0.875	1.045	0	7	

Regarding safety in car sharing, Yu et al. (2023) utilized a structural equation model to investigate “how the group representing relative advantages, environmental awareness, and perceived risk influences the other group, including green perceived usefulness and perceived ease of use, to result in the sharing of autonomous vehicles.” Specifically, perceived risk negatively influences green perceived usefulness and perceived ease of use, which reduces the intention to share autonomous vehicles [17]. Regarding socioeconomic issues related to road transport, Yu et al. (2024) employed ordered logit models with latent variables derived from structural equation models as independent variables. In terms of safety, we do not find a specific variable influencing car sharing. A higher income level increases the tendency to car-share, while being a woman and being elderly reduce this tendency [18].

This research makes a unique contribution by examining how the relative liability of participants, together the vehicle types involved, influences traffic accident injury severity. In addition, the study simultaneously analyzed 27 types of regulatory violations, 18 types of road transport accidents, 19 geographic features of road infrastructure, and several socioeconomic variables. This research fills an important gap in the literature by jointly analyzing relative liability, vehicle type, road structure, rule violations, accident types, and socioeconomic factors in a unified framework. To our knowledge, no prior study has integrated all of these dimensions when modeling traffic injury severity.

## Calculations

### Data management

Motor vehicle insurance data is one of the most appropriate sources for the integrated analysis of a nation’s road safety, which requires information about accident types, traffic rule violations, geographic road features, vehicle types, relative liability, and injury severity. Determining insurance rates requires insurance companies to analyze various factors influencing accident severity, such as violations, accident types, and local geography [19]. Moreover, insurance companies must interpret causal elements within the framework of relevant domestic traffic safety regulations, as their initial interpretation may result in regulatory actions or legal judgments from local jurisdictions. For instance, in the United States, fines are determined based on the weight of the vehicles involved, whereas in South Korea, fines are imposed according to the type of vehicle. Similarly, the penalty fees for speed limit violations in the United States are between 256% and 513% higher than those in South Korea [20].

Therefore, this study gathered data from major Korean motor vehicle insurance companies to conduct an integrated analysis aimed at identifying the primary factors contributing to road accidents. The data were coded in accordance with Korean transport regulations. The datasets included more specific components of road accident information than those employed in previous studies, which did not address the relative liability of involved parties or include all accident and traffic violation types specified in Korean regulations. The present study analyzed 27 types of rule violations, 18 types of accidents, and 19 geographic road features derived from photographic records of each accident. No prior study has considered all these variables.

This study analyzed the original accident records compiled by the Korean government involving 3,370 individuals from 1,800 road accidents that occurred between 2008 and 2013. Population density and the number of high schools were extracted from Korean census microdata, and transport GIS data were obtained from the Korea Transport Institute website. Variables were coded per individual, as a single accident may involve multiple persons whose injury severities can differ significantly. For example, a road accident may involve a driver—often considered primarily responsible for the crash—a pedestrian, or the occupants of another vehicle involved in the collision. The type of vehicle is a critical factor, as injury severity can vary considerably depending on the vehicle’s rigidity, even under identical accident or rule-violation conditions. To account for this complexity, this study coded the parties involved, their vehicle types, and their relative liability. For instance, in a collision between a passenger car and a motorbike where the motorbike driver is at fault, the motorbike driver (vehicle type: motorbike) is classified as “the person with higher liability,” whereas the car driver (vehicle type: passenger car) is identified as “the person with lower liability.” The original documents from the insurance companies specify the relative liability assigned to each party in every accident.

If the injury severity spans too many categories, the explanatory power of the logit model is reduced. Accordingly, the present study simplified the original dependent variable—number of hospitalization weeks—into three categories: “no injury,” “injury,” and “death.” These coding follows the criteria outlined in Chapter 11 of the Road Traffic Act and the Special Act on Traffic Accident Handling (revised on 28 July 2016). For detailed classification and coding, this study referenced GIAK [21].

Additionally, the structural features of the road at the accident site were classified based on the graphical illustrations in GIAK [21] and photographic descriptions included in each individual accident record. Exemplary categories included crosswalks with or without pedestrian signals, proximity to pedestrian sidewalks, and locations lacking a clear distinction between roadways, sidewalks, bike lanes, or overpass roads. Similar to the classification of violation types, there were instances in which an accident occurred in areas featuring multiple geographic road features. Regarding socioeconomic variables contributing to traffic accidents in Korea, this study considered only population density and the number of high schools per dong, following preliminary validity assessments. To represent categorical variables—such as accident types, rule violations, and road structure features—this study employed dummy variables (binary indicators). The year and region of each accident were included as additional control variables.

### Ethics statement

This study is a retrospective analysis of anonymized secondary data obtained from Korean motor vehicle insurance companies. All data were fully anonymized before access by the authors, and no personally identifiable information was accessible at any stage of the analysis. Therefore, institutional review board (IRB) approval and informed consent were not required. No minors were individually identified or involved, and no interaction with human participants occurred.

### Model specification and variable selection

Initially, the dataset contained more than 140 variables. Through stepwise ordered logit regression, variables that did not significantly contribute to the explanation of variance were removed. The criterion for the elimination was that the probability of the T value of the variable was higher than 0.2, which is a common standard for variable removal [22]. Notably, lowering this threshold below 0.2 considerably reduced the model’s explanatory power. Subsequently, the variance inflation factor (VIF) was examined to detect multicollinearity issues. According to Pennsylvania State University [23], if the *k*^th^ variable’s *R*^2^ value on the other predictors is *R*_*K*_^2^, the VIF of the *k*^th^ variable is expressed as


$$\text{VIF}\;=\;\frac{\text{1}}{\text{1}-{R_{K}}^{\text{2}}}.$$ (1)

Hair et al. recommended eliminating variables with a VIF greater than 10 [24], and this criterion was applied in the present study to exclude problematic variables. The average VIF of the remaining variables was 1.36, and the highest VIF was 3.59, indicating that multicollinearity was not present. The variables selected for analysis are listed in Table 1. The dependent variable is injury severity, categorized into three groups: no injury, injury, and death.

We deleted or merged confounding variables. Specifically, we examined all pairwise correlations among the variables and removed one variable from each pair with a correlation coefficient greater than 0.5. A similar procedure was applied to variables with a VIF greater than 4. While these procedures reduce multicollinearity and mitigate some confounding influences, unmeasured confounding cannot be fully ruled out due to the observational nature of the dataset.

We classified the variables according to Korean insurance criteria, which differ from those of Wang et al. [16]. Future research can adopt alternative classification schemes if more detailed information becomes available. Regarding socioeconomic variables, Yu et al. [25,26] provide a more detailed classification for future research.

This research adopted a generalized ordered logit model and an ordered logit model, and the results were compared. When the dependent variable has *M* categorical and ordered values, a GOLogit model takes the following form [27]:


$$P\left(Y_{i}>J\right)=\;\frac{\text{exp}(\alpha_{j}+X_{i}\beta_{j})}{\text{1}+[\text{exp}(\alpha_{j}+X_{i}\beta_{j})]},\;J=\text{1},\;\text{2},\;..,\;M-\text{1}.$$ (2)

In Equation (2), *P* represents the probability that the output variable is higher than *J*, which is the category number. *Xi* and *βi* denote the *i*^th^ independent variable and the corresponding coefficient (log-odds), respectively. In the context of this study, *Xi* includes explanatory variables such as vehicle type (e.g., bus, truck, motorcycle), rule violations (e.g., jaywalking, centerline crossing), road structure (e.g., number of lanes, intersection type), and socioeconomic indicators (e.g., age, population density). The coefficient *βi* represents the marginal change in the log-odds of transitioning to a higher severity category associated with each explanatory variable. Although the ordered logit model (O.L.M.) adopts the same general structure, its coefficients (*ß*) remain constant across all cumulative binary comparisons of the dependent variable’s categories—a limitation that does not reflect real-world data, as noted by Williams [27].

If the parallel regression assumption holds, the coefficient of a given variable should be the same across the series of logit models used in this study: one comparing “no injury” and “injury or death” and the other comparing “no injury or injury” and “death.” If this assumption is violated, a generalized ordered logit model would be more suitable. The conventional ordered logit model is a special case of the generalized version and is only appropriate when the parallel regression assumption is satisfied. In contrast, a multinomial logit model, which does not account for the ordinal nature of the dependent-variable categories—“no injury,” “injury,” and “death”—may not be appropriate [27].

When only a small number of variables violate the parallel regression assumption, it is preferable to release only those variables from the constraint. In such cases, the generalized ordered logit model is referred to as a partially ordered model [27]. Autofit—a STATA command—provides the iterative procedure that identifies such variables and frees them from parallel regression constraints. The result of the “Autofit” operation is detailed in Table 2, where the variable coefficients in the third column differ from those in the fourth.

**Table 2:** Results of the O.L.M. and the GOLogit model.

	Ordered logit model						Generalized ordered logit model						Generalized ordered logit model					
	Log-odds are equal through categories						“No injury” → “injury” or “death”						“No injury” or “injury” → “death”					
Variables	Coef.	Std. Err.	*Z*	*P* > *z*	[95% Conf.Interval]		Coef.	Std. Err.	*Z*	*P* > *z*	[95% Conf.Interval]		Coef.	Std. Err.	*z*	*P* > *z*	[95% Conf.Interval]	
Single-vehicle collision	1.346	0.284	4.74	0	0.790	1.903	1.775	0.315	5.63	0	1.158	2.393	1.775	0.315	5.63	0	1.158	2.393
Collision with a parked vehicle	0.899	0.241	3.72	0	0.425	1.372	0.958	0.250	3.83	0	0.467	1.448	0.958	0.250	3.83	0	0.467	1.448
Bus (more responsible)	1.004	0.137	7.35	0	0.736	1.271	1.759	0.255	6.91	0	1.260	2.258	1.063	0.172	6.19	0	0.726	1.399
Two-wheeler (more responsible)	–0.963	0.225	–4.28	0	–1.403	–0.522	–1.924	0.346	–5.57	0	–2.601	–1.247	0.259	0.412	0.63	0.53	–0.548	1.066
Collision at or near a crossroad	0.477	0.117	4.09	0	0.248	0.706	0.483	0.127	3.81	0	0.234	0.731	0.483	0.127	3.81	0	0.234	0.731
Truck (more responsible)	0.595	0.223	2.67	0.008	0.158	1.033	0.672	0.236	2.84	0.004	0.209	1.136	0.672	0.236	2.84	0.004	0.209	1.136
Bus (less responsible)	–2.832	0.284	–9.99	0	–3.388	–2.276	–3.437	0.348	–9.87	0	–4.120	–2.755	–1.740	0.463	–3.76	0	–2.647	–0.833
Two-wheeler (less responsible)	3.899	0.175	22.24	0	3.556	4.243	4.937	0.327	15.1	0	4.296	5.578	1.597	0.264	6.05	0	1.079	2.114
Bike with a motor (less responsible)	4.232	1.111	3.81	0	2.055	6.410	2.834	0.986	2.87	0.004	0.901	4.768	2.834	0.986	2.87	0.004	0.901	4.768
Bike without a motor (less responsible)	4.746	0.286	16.58	0	4.185	5.307	3.537	0.263	13.42	0	3.020	4.053	3.537	0.263	13.42	0	3.020	4.053
Agricultural machines (less responsible)	5.228	0.535	9.78	0	4.180	6.277	3.512	0.510	6.89	0	2.513	4.511	3.512	0.510	6.89	0	2.513	4.511
Pedestrian (less responsible)	4.912	0.237	20.74	0	4.447	5.376	7.336	1.029	7.13	0	5.318	9.354	2.923	0.285	10.25	0	2.364	3.482
Jaywalker (less responsible)	5.099	0.236	21.64	0	4.637	5.561	6.532	0.757	8.63	0	5.049	8.015	2.959	0.273	10.84	0	2.424	3.494
Passenger (less responsible) (vehicle occupant, excluding driver)	3.433	0.153	22.47	0	3.133	3.732	6.630	0.722	9.18	0	5.214	8.045	0.924	0.223	4.15	0	0.487	1.361
On a speedbump or a curved road	3.111	1.615	1.93	0.054	–0.055	6.276	3.407	1.548	2.2	0.028	0.373	6.442	3.407	1.548	2.2	0.028	0.373	6.442
Number of lanes on the road	0.198	0.045	4.45	0	0.111	0.285	0.162	0.056	2.89	0.004	0.052	0.271	0.347	0.073	4.78	0	0.205	0.490
Violation of the speed limit	1.245	0.462	2.69	0.007	0.339	2.152	0.842	0.541	1.56	0.12	–0.219	1.903	2.456	0.594	4.14	0	1.292	3.619
Violation of slowing down or stopping	–0.852	0.350	–2.43	0.015	–1.538	–0.166	–0.942	0.433	–2.17	0.03	–1.791	–0.093	–0.942	0.433	–2.17	0.03	–1.791	–0.093
Crossing the centerline	0.955	0.181	5.28	0	0.600	1.309	0.855	0.208	4.11	0	0.448	1.262	1.442	0.267	5.4	0	0.919	1.965
Not adhering to the priority order	–0.381	0.145	–2.64	0.008	–0.665	–0.098	–0.414	0.159	–2.61	0.009	–0.726	–0.103	–0.414	0.159	–2.61	0.009	–0.726	–0.103
Failure to maintain a watch for safe driving	–0.401	0.120	–3.35	0.001	–0.636	–0.167	–0.615	0.148	–4.14	0	–0.906	–0.324	–0.023	0.182	–0.13	0.898	–0.379	0.333
Driving drunk or asleep and crossing the centerline	1.942	0.832	2.33	0.02	0.311	3.573	1.921	0.738	2.6	0.009	0.474	3.367	1.921	0.738	2.6	0.009	0.474	3.367
Age	0.004	0.003	1.23	0.218	–0.002	0.010	–0.005	0.005	–0.98	0.325	–0.015	0.005	0.012	0.005	2.54	0.011	0.003	0.021
Population density	0.000	0.000 (–1.055)	–5.11	0	0.000	0.000	0.000	0.000 (–3.092)	–3.2	0.001	0.000	0.000	0.000	0.000	–6.03	0	0.000	0.000
1 a.m.	0.742	0.252	2.94	0.003	0.247	1.236	0.737	0.258	2.86	0.004	0.232	1.242	0.737	0.258	2.86	0.004	0.232	1.242
5 a.m.	0.534	0.231	2.31	0.021	0.081	0.988	0.573	0.239	2.39	0.017	0.104	1.043	0.573	0.239	2.39	0.017	0.104	1.043
3 p.m.	–0.461	0.208	–2.21	0.027	–0.869	–0.052	–0.079	0.275	–0.29	0.774	–0.618	0.460	–1.629	0.495	–3.29	0.001	–2.599	–0.659
/cut1	1.927	0.252			1.432	2.421	–1.468	0.335	–4.38	0	–2.124	–0.811	–5.461	0.414	–13.19	0	–6.273	–4.650
/cut2	6.464	0.290			5.895	7.033												
	Number of obs. = 3,370						Number of obs. = 3,370											
	LR chi^2^(57) = 2563.8						LR chi^2^(77) = 2972.06											
	Prob > chi^2^ = 0						Prob > chi^2^ = 0											
	Pseudo-R^2^ = 0.4124						Pseudo-R^2^ = 0.4781											
	Log-likelihood = –1826.356 (Initial value) = –3108.257						Log-likelihood = –1622.226											
	Approximate likelihood-ratio test of proportionality of odds across response categories: chi^2^(54) = 518.36						Wald test of parallel lines assumption for the final model: chi^2^(37) = 27.16											
	Prob > chi^2^ = 0.0000						Prob > chi^2^ = 0.8820											

Table 2 summarizes the results from the GOLogit and O.L.M., showing the coefficients of only the statistically significant variables, along with the standard errors and the z-value and p-value. The first column shows the independent variables, the second column shows the result of the O.L.M., and the third and fourth columns explain the two binary logit models of the GOLogit model. The second column applies the parallel lines assumption that all the coefficients of a variable in the two binary logit models are equal. The third column’s model shows the log-odds of each variable from “no injury” to “injury or death” while the fourth shows the corresponding values of each variable from “no injury or injury” to “death.” STATA’s Autofit option did not apply the parallel assumptions for the following variables in the third and fourth columns:

**Table:** 

(1) Bus (more responsible);	(2) Two-wheeler (more responsible);
(3) Jaywalker (more responsible);	(4) Bus (less responsible);
(4) Truck (less responsible);	(5) Two-wheeler (less responsible);
(6) Pedestrian (less responsible);	(7) Jaywalker (less responsible);
(8) Passenger (less responsible);	(9) Junction of several roads;
(10) Number of lanes on the road;	(11) Crossing the centerline;
(12) Failure to maintain a watch for safe driving;	(13) Age of the party
(14) Population density;	(15) 12 a.m. and 3 p.m.

We applied technical methods to enhance the explanatory power of the model, e.g., screening variables based on correlation coefficients, investigating the VIF, including cities as control variables, and stepwise regression with a threshold of 0.2. Therefore, the pseudo-R-squared increased from <0.3 to approximately 0.48, indicating that the procedure was exploratory, aimed at finding an optimal model specification rather than a strictly calculated solution. This is illustrated by the variable “age,” which had a coefficient of 0.218 in the ordered logit model but 0.001 in the generalized ordered model (Table 2). The threshold of 0.2 was chosen to avoid prematurely excluding variables with potential substantive relevance, given the exploratory nature of our modeling strategy. Additional tests using an alternative threshold of 0.1 yielded a similar set of retained variables, indicating that our main findings are not sensitive to the specific threshold choice.

## Results

The GoLogit model exhibited several functional advantages over the O.L.M. First, its log-likelihood exceeded that of the O.L.M. (–1622.23 vs. –1826.36), indicating that it has higher accuracy than the O.L.M. Second, its log-likelihood of chi-squared was more statistically significant than that of the O.L.M. (2972.06 vs. 2563.8). Finally, its pseudo-R2 (0.4781 vs. 0.4124), which measures the model’s goodness of fit, exceeded that of the O.L.M. The pseudo-R2 of this model was higher than that of some models used in previous studies. For example, the improvement in the pseudo-R2 from a fixed- to a random-parameter logit model was 2.4% to 2.9% in the study of Anastasopoulos and Mannering [11]. However, in the present study, it was enhanced by 15.9% (0.4781 vs. 0.4124). Regarding the parallel lines assumption, the O.L.M. presented in the second column of Table 2 violated this assumption significantly (see the bottom row of the table), justifying the use of the GoLogit model. For example, the variable “collision at or near a crossroad” yielded significantly positive log-odds for higher injury severity in all cumulative binary comparisons. Stepwise analysis indicated that this variable increased the likelihood of injury severity being (1) “injury or death” as opposed to “no injury” and (2) “death” as opposed to “no injury or injury.” The GoLogit model calculates the log-odds for (1) and (2) separately.

“Collision at junctions, where several roads, sidewalks, or bike lanes meet” exhibited positive and significant log-odds of higher injury severity in the O.L.M., whereas in the GoLogit model, significance was observed only in the comparison between “no injury or injury” and “death.” The log-odds of “a collision between a vehicle and an unmovable structure,” such as a building, were also positive and significant (see: single-vehicle collision). Similarly, significant results were found for incidents in which a vehicle struck a stationary, parked car, caused by the driver’s insufficient vehicle control skills. Additionally, this study estimated positive and significant log-odds for accidents involving problematic road structures. For example, the log-odds of a “combination of a speedbump and a curved road” were significantly positive at 3.407 (compared to 3.111 in the O.L.M.), exceeding those for “a junction of several roads” (2.189 in GoLogit and 1.080 in O.L.M.). Likewise, “a road having several lanes” exhibited positive log-odds, possibly because such road types increase the likelihood of pedestrian exposure while crossing or increase the risk of broadside vehicle collisions.

Regarding traffic rule violations, “violating the speed limit” produced positive log-odds of higher injury severity in the comparison between “no injury or injury” and “death.” The log-odds in the second category (non-death to death), at 2.456, were higher than those in the first category (non-injury to injury or death), at 0.842. This indicates that the likelihood of fatal injury is higher than that of non-fatal injury when a driver exceeds the speed limit. The combined violations of “driving while intoxicated or asleep and crossing the centerline” and “crossing the centerline with unclear lane demarcation” increased the log-odds in both categories in both the GoLogit model and the O.L.M. In summary, committing multiple violations simultaneously elevated the likelihood of sustaining more severe injuries. Contrary to common expectations, violations such as “failure to maintain a proper lookout for safe driving” or “not adhering to priority rules” did not appear to be significantly dangerous, even though the OECD identifies them as the most frequent violations [1]. Therefore, this study concludes that the frequency of certain types of traffic violations is not necessarily aligned with the severity or magnitude of injury resulting from a single incident.

The vehicle types, coded along with relative negligence, also yielded notable results. When a heavier vehicle, such as a bus or a truck, was more responsible for an accident, this study observed higher injury severity compared to cases involving lighter vehicles. However, when the heavier vehicle (e.g., a bus) was less responsible, the log-odds decreased, suggesting that a lighter impact from the opposing party on a heavy vehicle did not result in serious injury. Furthermore, significant log-odds of higher injury severity were found when a lighter vehicle—such as a bicycle or pedestrian—was less responsible for an accident, indicating that an unsuspecting lighter party struck by a heavier vehicle tended to sustain more severe injuries. Another distinctive finding was that passengers with less liability exhibited higher log-odds of sustaining severe injuries. This pattern appears to result from frequent collisions between passenger cars and their passengers, typically caused by driver negligence.

A significant socioeconomic observation was that population density substantially reduced the log-odds of sustaining severe injuries. The absolute magnitude of the odds is shown in parentheses in the row corresponding to “population density” (Table 2). To ensure comparability with other binary independent variables, the original odds were scaled upward by multiplying them by the maximum value of population density. The results are consistent with Cho’s interpretation that higher population density may reflect a more advanced urban infrastructure, offering better protection for transportation users [28]. Another noteworthy finding was that older individuals were more likely to suffer severe injuries. This is consistent with the OECD’s warning that Korea could see an increase in elderly victims due to urban designs that are less pedestrian-friendly, particularly as the aging population faces growing poverty and is increasingly compelled to walk [1]. What distinguishes this study from prior research [1] is its empirical validation of these observations. Regarding the regional economy, Korea displays several prominent trends consistent with the socioeconomic patterns described above. For instance, the density of manufacturing companies tends to be inversely related to income levels [29], while the interaction between “sales within the same industries” and “accessibility to public transport” is positively associated with house prices [30]. In contrast, other industries exhibit the opposite pattern. We initially reviewed additional socioeconomic variables such as household income, Gini coefficients, and vehicle ownership. However, these variables were excluded due to limitations in data availability and granularity at the required spatial resolution. Including these socioeconomic variables without appropriate spatial matching would have introduced additional bias into the model, so we excluded them to maintain specification validity. Therefore, only population density and the number of high schools were retained as reliable indicators.

## Discussion

Our findings are broadly consistent with previous crash-severity studies that emphasize the heightened vulnerability of pedestrians and cyclists. For example, Rolison et al. (2018) reported that pedestrian-involved crashes yield disproportionately severe outcomes, a pattern mirrored in our GOLogit estimates showing particularly high log-odds for pedestrians and non-motorized bicycle users [5]. Likewise, Savolainen et al. (2011) highlighted the importance of accounting for within-crash factors such as vehicle type and responsibility; our incorporation of relative liability extends this line of work by empirically demonstrating that less-responsible parties in weaker vehicle categories face substantially higher injury risks [15]. Prior studies using ordered logit and probit models have also noted the limitations of the parallel-regression assumption, and our results confirm these concerns, supporting the use of a generalized ordered logit structure to better capture heterogeneous severity effects across injury categories.

Taken together, these empirical patterns reinforce the broader consensus in prior studies while extending the literature through our explicit treatment of relative liability and contextual road-infrastructure factors. Compared with prior studies, our analysis provides several distinct extensions. While Wang et al. (2023) focused on motorcycle-involved crashes using a partial proportional odds model, our study expands the analytical scope by incorporating a broader range of road users—including pedestrians, cyclists, and different vehicle categories—and by jointly modeling road-structure variables, rule violations, and socioeconomic factors [16]. In addition, Savolainen et al. (2011) emphasized the importance of within-crash correlations but noted that most studies consider only the most severely injured party [15]. By contrast, our model incorporates the relative liability of both involved parties, empirically demonstrating asymmetric injury risks for less-responsible and structurally weaker road users. Finally, although Yu et al. (2023, 2024) examined attitudinal and demographic influences using behavioral survey data [17,18], our study complements this line of research by applying the GOLogit model to real-world accident records, revealing how socioeconomic vulnerability and road-infrastructure conditions interact to shape injury severity. These empirical patterns directly inform the following policy implications.

This study suggests the following policy recommendations for road traffic safety in Korea based on the results:

First, these findings indicate that Korea should reinforce its policies to protect pedestrians. The log-odds were substantially higher for pedestrians (including jaywalkers). Lowering speed limits in densely populated residential areas should be prioritized, as it can directly reduce injury severity by decreasing the impact force of collisions. In this context, it is also recommended that the construction of wide roads be limited unless supported by a robust feasibility assessment, as such roads increase pedestrian exposure to vehicular collisions.

Second, policy development should account for the finding that the party with lower liability often sustains more severe injuries when associated with structurally weaker vehicles. In contrast, even pedestrians with greater liability did not exhibit significantly higher log-odds of injury severity. To mitigate the disproportionate harm resulting from minor, often unrecognized errors, it is suggested that advanced technologies—such as predictive braking systems—be implemented to halt vehicles just before collisions involving vulnerable users.

Third, the Korean government must improve road infrastructure. For instance, the presence of a speedbump on a curved road can exacerbate injury severity. Traditional intersections, such as right-angled crossroads, should be redesigned—expanding the use of roundabouts may be beneficial, as they naturally slow or stop incoming traffic. Enhancing the visibility of lane markings through repainting or other means could also reduce the severity of accidents.

Fourth, the enforcement of core traffic regulations—particularly those concerning speed limits and crossing centerlines—should be intensified. This may involve increasing human resources or deploying automated systems to monitor compliance. Additionally, this study highlights the need for improved traffic safety programs and regulations targeted at elderly road users. Lastly, public resources should not be overallocated to improve safety in areas already considered safe. As indicated by our finding that higher population density correlates with a reduced likelihood of injury, densely populated cities may already benefit from sufficient safety mechanisms.

Although this study focuses on road traffic, the methodological insights—particularly the treatment of heterogeneous severity mechanisms—are transferable to other safety-critical domains. This framework also supports the Korean R&D project “Development of Technology to Analyze, Evaluate and Prevent Human Errors of Railroad Workers” in evaluating railroad operation-related risks in an integrated manner.

## Conclusions

The GOLogit model, which was uniquely applied to road safety analysis in this study, demonstrated superior explanatory power compared to the conventional ordered logit model and alternative logit models used in previous studies. Another methodological contribution of this study is the incorporation of combined information on vehicle type and relative liability, allowing the model to distinguish heterogeneous injury risks between more- and less-responsible parties across different vehicle categories. Through this innovative approach, we found that pedestrians with minimal responsibility for an accident were the most vulnerable, whereas those more accountable for the collision were significantly safer. This finding underscores the need for heightened driver awareness and reinforces the necessity for stricter enforcement of pedestrian protection policies. Additionally, this study highlights that speed violations and centerline crossing remain persistent issues, necessitating more vigorous regulatory enforcement. However, contrary to common assumptions, the likelihood of sustaining severe injuries was lower in highly developed areas with dense populations. This suggests that allocating road safety investments disproportionately to wealthier regions may not be the most effective strategy, emphasizing the need for equitable distribution of resources to improve overall traffic safety in Korea.

Future research should expand on this work. Specifically, analyses using more recent data (since 2013) are necessary to reflect Korea’s latest urban developments and policy adjustments. Our findings are based on insurance records from 2008 to 2013, which, although broad in scope, may not fully capture recent shifts in traffic safety trends or behavior. These limitations should be taken into account when interpreting the results. Moreover, including additional socioeconomic factors, such as household income or vehicle ownership rates, would deepen the understanding of vulnerability and liability. Lastly, addressing unobserved variables or applying causal inference methods could strengthen the robustness of our conclusions. As with all observational studies, some residual confounding may remain, and future research applying causal inference methods may help validate these findings. While minor and uninsured crashes might be underreported, significant traffic accidents—particularly those resulting in severe injuries or fatalities—are typically covered by insurance claims, ensuring that our data reflects the most policy-relevant incidents.
